# Mangrovimonas cancribranchiae sp. nov., a novel bacterial species associated with the gills of the fiddler crab Cranuca inversa (Brachyura, Ocypodidae) from Red Sea mangroves

**DOI:** 10.1099/ijsem.0.006415

**Published:** 2024-06-12

**Authors:** Xinyuan Yang, Elisa Garuglieri, Marc W. Van Goethem, Ramona Marasco, Marco Fusi, Daniele Daffonchio

**Affiliations:** 1 Red Sea Research Center (RSRC), Biological and Environmental Sciences and Engineering Division (BESE), King Abdullah University of Science and Technology (KAUST), Thuwal, 23955-6900, Saudi Arabia; 2 Dove Marine Laboratory, School of Natural and Environmental Sciences Newcastle University, Newcastle-Upon-Tyne, NE1 7RU, UK

**Keywords:** *Mangrovimonas*, symbionts, gills, mangrove, *Bacteroidota*, brachyuran crabs, *Cranuca inversa*

## Abstract

Two bacteria, UG2_1^T^ and UG2_2, were isolated from the gill tissues of the mangrove fiddler crab *Cranuca inversa* collected on the east coast of the Red Sea (Thuwal, Saudi Arabia). The cells are Gram-negative, rod-shaped, orange-pigmented, motile by gliding with no flagella, strictly aerobic, and grow at 20–37 °C (optimum, 28–35 °C), at pH 5.0–9.0 (optimum, pH 6.0–7.0), and with 1–11 % (w/v) NaCl (optimum, 2–4 %). They were positive for oxidase and catalase activity. Phylogenetic analysis based on 16S rRNA gene sequences indicated that isolates UG2_1^T^ and UG2_2 belong to the genus *Mangrovimonas*, showing the highest similarity to *Mangrovimonas spongiae* HN-E26^T^ (99.4 %). Phylogenomic analysis based on the whole genomes, independently using 49 and 120 concatenated genes, showed that strains UG2_1^T^ and UG2_2 formed a monophyletic lineage in a different cluster from other type strain species within the genus *Mangrovimonas*. The genome sizes were 3.08 and 3.07 Mbp for UG2_1^T^ and UG2_2, respectively, with a G+C content of 33.8 mol% for both strains. Values of average nucleotide identity and digital DNA–DNA hybridization between the strains and closely related species were 91.0 and 43.5 %, respectively. Chemotaxonomic analysis indicated that both strains had iso-C_15 : 0_ and iso-C_15 : 1_ G as dominant fatty acids, and the primary respiratory quinone was identified as MK-6. The major polar lipids comprised phosphatidylethanolamine, one unidentified glycolipid, one unidentified phospholipid, two unidentified aminolipids, and four unidentified lipids. Based on phylogenetic, phylogenomic, genome relatedness, phenotypic, and chemotaxonomical data, the two isolates represent a novel species within the genus *Mangrovimonas*, with the proposed name *Mangrovimonas cancribranchiae* sp. nov., and the type strain UG2_1^T^ (=KCTC 102158^T^=DSM 117025^T^).

## Introduction

**Fig. 1.**
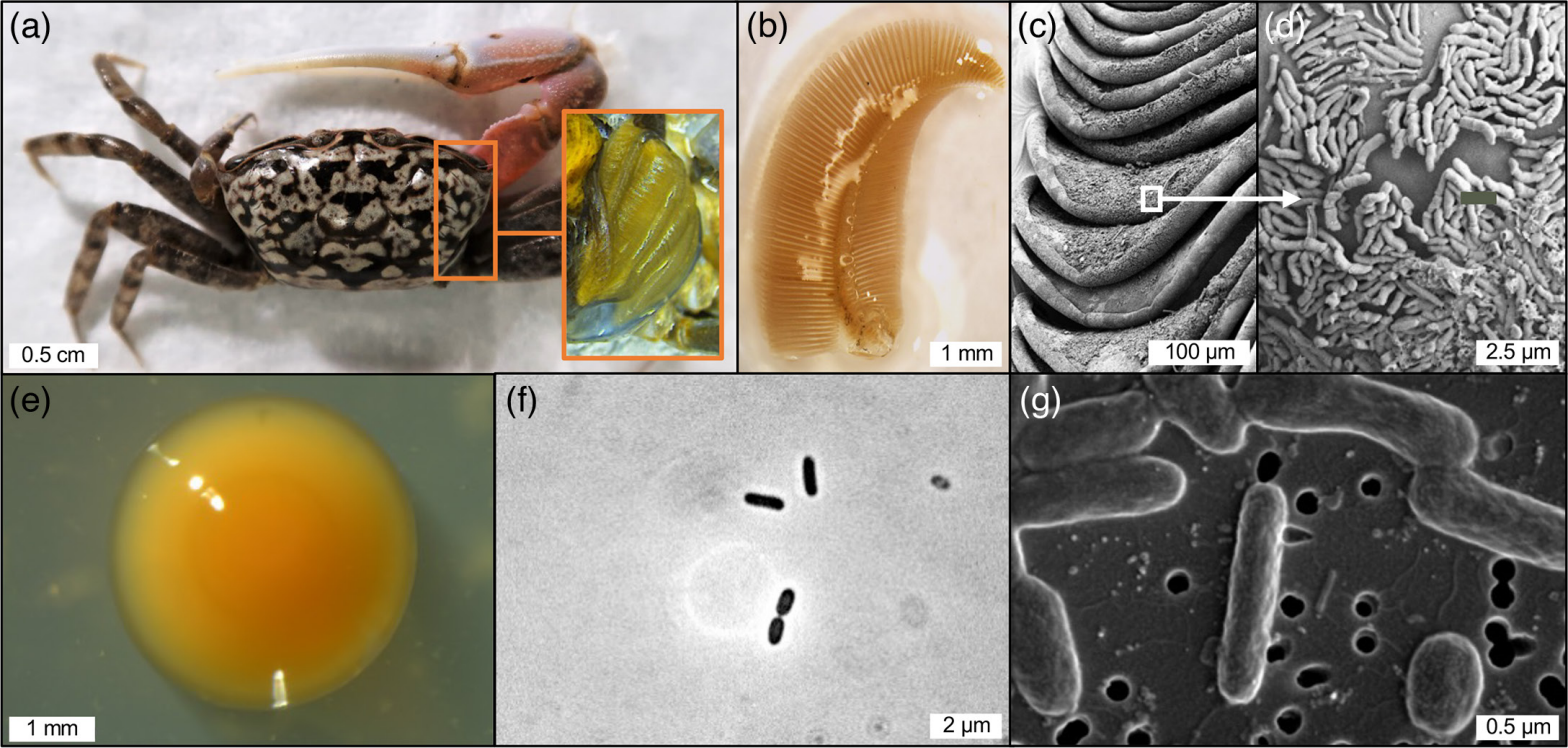
*Cranuca inversa*’s gill microenvironment and morphological features of the novel strains. (**a**) A male individual of *Cranuca inversa* before the dissection; the box shows the right gills exposed after the carapace removal. (**b**) A single dissected gill of *C. inversa* under the stereomicroscope. (**c**) Microscope images of *C. inversa* gill lamellae and (**d**) the associated microbiome taken under a teneo scanning electron microscope (FEI); the gill tissue specimen was fixed, prepared and imaged as described by Garuglieri and colleagues [11]. (**e**) UG2_1^T^ colony observed under the stereomicroscope, and (**f and g**) its cells under the optical microscope and scanning electron microscope, respectively.

Brachyuran crabs are an essential component of the macrobenthic biomass present in mangrove ecosystems, exhibiting extensive evolutionary radiation and successfully colonizing a broad range of diverse ecological niches [1]. Among Brachyura, fiddler crabs (family *Ocypodidae*) are bimodal animals inhabiting the intertidal zone of mangrove forests [2,3]. Their daily activities as sediment bioturbators [4,6] imply a consistent transit of these animals among aquatic environments, anoxic/sulphidic sediments, and open air. To cope with the challenges posed by such a lifestyle, they have evolved physiological [7], morphological [8] and behavioural [9] adaptations, including structural modifications of their gills, which are key organs in the evolutionary adaptation of crabs. Recently, microbial communities associated with the gills of mangrove fiddler crabs have been described [10,14], garnering attention for their potential symbiotic and coevolutionary implications. In particular, the gill-associated microbiomes of *Cranuca inversa* (Fig. 1a–d) have been proposed to help the animal in the disposal of ammonia (the primary nitrogenous waste of crabs) during periods of exposure to air and to protect it from other potentially toxic compounds, such as hydrogen sulphide and carbon monoxide [12]. This relationship supports the concept of a true symbiosis that could have contributed (and still does) to the adaptation of these crab species to intertidal environments and, to a broader extent, to the terrestrialization of these animals [11,12, 14, 15].

Despite the progress achieved with molecular methods in the investigation of the mangrove crab symbiotic microbiome, the isolation of cultivable strains from crab organs, such as gills, remains overlooked. In this context, we applied a cultivation approach based on differential centrifugation to isolate bacteria associated with *C. inversa* gills. Among the purified isolates, we recorded two isolates that could not be assigned unequivocally to any validly identified type strain within the genus *Mangovimonas* (family *Flavobacteriaceae*). At the time of writing, the genus *Mangrovimonas* comprises four species with validly published names, namely *Mangrovimonas yunxiaonensis* [16], *Mangrovimonas spongiae* [17], *Mangrovimonas futianensis* [18], and *Mangrovimonas aestuarii* [19], and one invalidly published species *Mangrovimonas xylaniphaga* [20]. All these species have a marine origin, particularly from mangrove ecosystems. Here, we describe the genomic, physiological, and metabolic features of two strains, UG2_1^T^ and UG2_2, as part of a novel *Mangovimonas* species for which the name *Mangrovimonas cancribranchiae* sp. nov. is proposed according to their origin from the gill of the mangrove crab *C. inversa*.

## Sampling and isolation

Male individuals of the fiddler crab *C. inversa* were collected in the *Avicennia marina* mangrove forest of Ibn Sina Field Research Station (22° 20′ 23.8″ N 39° 05′ 20.7″ E) at the King Abdullah University of Science and Technology (KAUST), Saudi Arabia in December 2019. The gills of eight crab individuals were pooled (fresh weight, ~0.3 g), manually homogenized with pestles (Eppendorf) in a 1.5 ml tube, and finally diluted in 5 ml of filtered-sterilized (0.2 µm pore size filters, VWR) seawater. The subsequent isolation strategy was designed to separate the bacteria closely from those loosely attached to the crab gill surface by performing differential centrifugations of the gill homogenate, as described by O’Connor and colleagues [21]. In brief, 2 ml gill homogenate was centrifuged at 284 *g* for 5 min at 4 °C. The supernatant was then transferred to a second tube and centrifuged again at 640 *g* for 5 min at 4 °C. The pellets from both centrifugation steps were pooled and resuspended in 1 ml sterile seawater. Primary mixed cultures were obtained by spreading aliquots (100 µl) of pellet and supernatant samples on three Zobell marine agar 2216 (MA; HiMedia) plates each (total *n*=6). The cultures were then incubated at 28 °C (based on the average environmental temperature at the time of sample collection) for 7 days. After the incubation, colonies of different morphologies were isolated by picking and transferring them onto fresh MA plates, obtaining the first isolate generation. Three consecutive generations were cultured to ensure the isolate strains’ purity by streaking a single colony of each solid culture on a new MA plate. At the third generation, one colony of each pure strain was transferred to 4 ml Zobell marine broth 2216 (MB; HiMedia) and incubated at 28 °C. The purity of liquid cultures was assessed through optical microscope observations and by plating the culture on MA plates to check for feature consistency. Aliquots of the pure microbial culture were mixed with the same volume (750 µL) of 50 % glycerol sterile solution and stored at –80 °C. For further analysis, the strains were routinely grown starting from 10 µl inocula of cryopreserved glycerol stocks using the same isolation media and conditions.

## 16S rRNA gene phylogenetic analysis

The genomic DNA was extracted from single colonies of isolates resuspended in 60 µl sterile seawater by heat treatment, as described by Dashti *et al.* [22]. The nearly full-length 16S rRNA gene sequence was amplified through PCR using the universal primers 27F (5′-AGAGTTTGATCATGGCTCAG-3′) and 1492R (5′-TACGGTTACCTTGTTA-CGACTT-3′). The PCR products were sequenced by the KAUST Bioscience Core Lab (Thuwal, Saudi Arabia) through Sanger sequencing. Following nucleotide trimming based on quality with the software Bioedit 7.2 [23] and Chromas 1.7 (Technelysium), forward and reverse reads were assembled into consensus sequences (~1400 bp fragments) and compared with the EzBioCloud 16S database (https://help.ezbiocloud.net/ezbiocloud-16s-database) [24] and using the blast tool against both Standard and rRNA/ITS GenBank databases (https://blast.ncbi.nlm.nih.gov/Blast.cgi; NCBI) [25].

During the initial screening in December 2019, two isolates from our collection, UG2_1 and UG2_2, were found to be related to *Mangrovimonas* but could not be identified without ambiguity to any validly described species. At that time, the closest relative type strain was *M. yunxiaonensis* LYYY01^T^, showing nucleotide identities of 95.6 and 95.4 % based on blast and EzBioCloud analyses, respectively, followed by *M. aestuarii* MBT5^T^ (95.2 % on blast) and *M. futianensis* AS18^T^ (94.8 % on blast and 94.9 % on EzBioCloud). In 2020, a novel type strain, *M. spongiae* HN-E26^T^, presenting a higher nucleotide identity (99.4%) with our isolates, was deposited in the LPSN. However, despite the 16S rRNA gene sequences of UG2_1 and UG2_2 exceeding the identity percentage thresholds with strain HN-E26^T^ for delineating a new species, phylogenetic analyses based on the 16S rRNA gene, as well as phylogenomic and genomic analyses described below, supported that the two isolates represent a novel species of *Mangrovimonas*, distinct from *M. spongiae* HN-E26^T^.

**Fig. 2.**
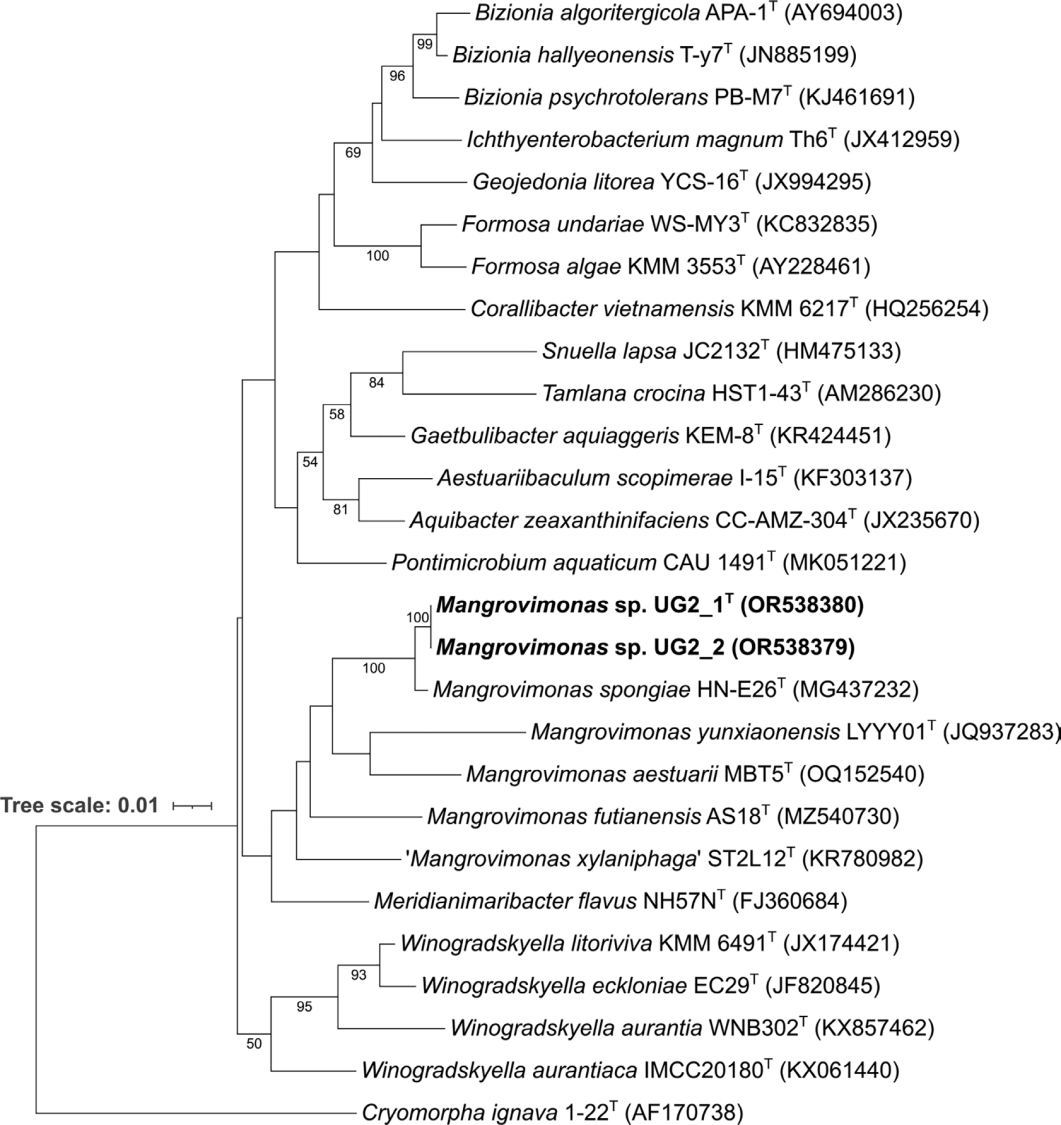
Maximum-likelihood phylogenetic tree based on 16S rRNA gene sequences and reconstructed using megaX version 11.0.10 [26], showing the relationships among UG2_1^T^, UG2_2 and close relative species in the family *Flavobacteriaceae*. UG2_1^T^ and UG2_2 are shown in bold. *Cryomorpha ignava* 1-22^T^ (AF170738) was used as the outgroup. The NCBI accession numbers are given after the species name. Numbers at the nodes are percentages of bootstrap values based on 1000 resampled datasets. Bar represents 0.01 substitutions per nucleotide position.

Phylogenetic trees based on the 16S rRNA gene sequence of the two isolates and their closest relative type species were reconstructed in megaX version 11.0.10 [26] by using the maximum-likelihood [27], neighbour-joining [28], minimum-evolution [29] and maximum-parsimony algorithms [30] with bootstrap values (1000 replications), based on the algorithm of Kimura’s two-parameter model [31]. *Cryomorpha ignava* 1-22^T^ (AF170738) was used as an outgroup species. In all the resulting phylogenetic trees, strains UG2_1 and UG2_2 clustered with *M. spongiae* on the same branch of the genus *Mangrovimonas* but formed an independent lineage, suggesting that they represent a yet undescribed species (Figs. 2, S1, S2 and S3 available in the online version of this article). Since the nearly full-length 16S rRNA gene sequences of UG2_1 and UG2_2 (GenBank OR538380 and OR538379) shared almost 100 % similarity, the two strains can be considered to belong to the same species [32]. Notably, the enterobacterial repetitive intergenic consensus (ERIC) PCR fingerprint [33] supported that UG2_1 and UG2_2 are clonal varieties (Fig. S4). Here, we propose UG2_1^T^ as the type strain of such a clade.

## Phylogenomics and genome analysis

The genomic DNA of UG2_1^T^ and UG2_2 isolates was extracted using a Maxwell RSC device (Promega) with cultured cell cartridges following the manufacturer's instructions. The extracted DNA was quantified using the Qubit 3.0 fluorometer with the Qubit dsDNA BR assay kit (Thermo-Fisher Scientific). Quality was checked by electrophoresis on 1.2 % agarose gels and via spectrophotometry (Nanodrop One spectrophotometer, Thermo-Fisher Scientific). Samples were sequenced at the KAUST Bioscience Core Labs using a PacBio CLR Sequel I (Pacific Biosciences). The obtained genome sequences have been deposited to GenBank with the accession numbers CP136925 and CP136924 for UG2_1^T^ and UG2_2, respectively. Raw genomic sequences were assembled through the Canu version 2.2 [34] pipeline using default parameters and specifying a predicted genome size of 4 Mbp. Genome quality was evaluated with quast version 5.2.0 [35] while completeness and contamination were determined with CheckM version 1.1.3 [36]. The assembled genomes were then annotated with Prokka version 1.14.6 [37] and were assigned taxonomy with GTDB-Tk version 1.3.0 [38], and were further analysed within KBase (https://www.kbase.us) [39]. We used geNomad (version 1.7) to determine the presence of plasmids and prophages in the genomes [40]. The prediction of carbon sources used by the strain based on the genome was made using the GapMind software [41]. Secondary metabolism was determined by predicting biosynthetic gene clusters using antiSMASH version7.0 [42].

The general parameters of the genomes are summarized in Table S1 and thoroughly described, together with the key results of annotated genomic features, below.

For the genome-based phylogenetic analysis, a phylogenomic tree based on the full genomes was reconstructed through a whole genome-based taxonomic analysis with the Type (Strain) Genome Server (TYGS; https://tygs.dsmz.de) [43,44]. The closest type strain genomes were determined against all TYGS type strain genomes via the mash algorithm [45]. Ten closely related type strains were also determined via 16S rRNA gene sequences using RNAmmer [46] and were used in blast queries [47] against the 16S rRNA gene sequences of TYGS type strains. The resulting best 50 matching type strains (according to the bitscore) for each genome were used to calculate phylogenomic distances using the Genome blast Distance Phylogeny approach (GBDP) under the algorithm ‘coverage’ and distance formula *d_5_* [48]. The resulting intergenomic distances were used to infer a balanced minimum-evolution tree with branch support via FastME 2.1.6.1, including SPR post-processing [49]. Branch support was inferred from 100 pseudo-bootstrap replicates each. Two additional phylogenomic trees were reconstructed by extracting 49 and 120 concatenated single-copy conserved marker genes from the genomes of UG2_1^T^, UG2_2, and closely related strains using FastTree2 [50] and GTDB-Tk [38], respectively. The trees were reconstructed based on the multiple sequence alignment of both these gene groups using a maximum-likelihood algorithm.

**Fig. 3.**
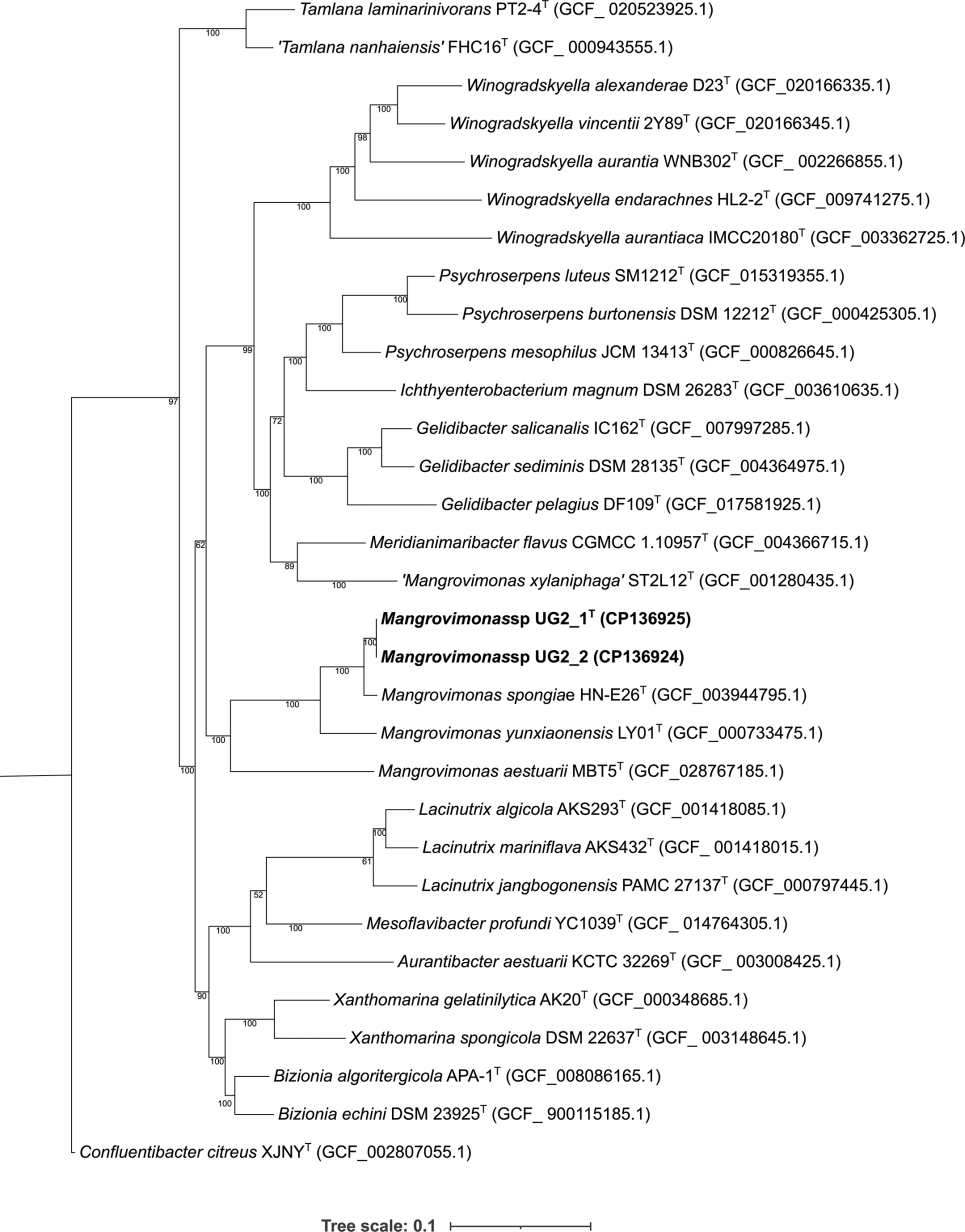
Phylogenetic tree with proposed strains UG2_1^T^, UG2_2 and related species based on 120 concatenated single-copy genes using the maximum-likelihood method made using the software GTDB-Tk [38]. The NCBI accession numbers are given after the species name. The tree is drawn to scale, with branch lengths measured in the number of substitutions per site. Numbers at nodes indicate bootstrap percentages using 1000 replicates. UG2_1^T^ and UG2_2 are shown in bold. *Confluentibacter citreus* XJNY^T^ (GCF_002807055.1) was used as the outgroup. Bar, 0.1 substitutions per site.

All the obtained phylogenomic trees (Figs. 3, S5 and S6) showed that UG2_1^T^ and UG2_2 formed a monophyletic lineage with a 100 % bootstrap value and represent a different species compared to the closest relative type species *M. spongiae* and the other type species within the genus *Mangrovimonas*.

To support the identification of the two isolated strains as belonging to a novel species, the digital DNA–DNA hybridization (dDDH), the average nucleotide identity based on reciprocal best hits (ANI), blast (ANIb) and MUMmer (ANIm) algorithms, and the average amino acid identity (AAI) between UG2_1^T^, UG2_2 and the four closest related strains were calculated and reported in Table S2. The dDDH values were obtained using the Genome-to-Genome Distance Calculator version 3.0 (https://ggdc.dsmz.de/ggdc.php) [48]. ANI values were calculated using the tool FastANI (https://bio.tools/fastani) [51], while ANIb and ANIm were determined using JSpeciesWS (https://jspecies.ribohost.com/jspeciesws) [52]. AAI was calculated using the tool AAI calculator (http://enve-omics.ce.gatech.edu/aai) [53].

The comparison between UG2_1^T^ and UG2_2 showed dDDH and ANI values of 100%, which are both above the recommended thresholds for prokaryotic species delineation (70 % for dDDH and 95 % for ANI) [52,54,57], confirming that they belong to the same species. Compared with the two closest related strains, *M. spongiae* HN-E26^T^ and *M. yunxiaonensis* LYYY01^T^, UG2_1^T^ and UG2_2 showed values of dDDH (both strains 43.5 % with *M. spongiae* and 20.8 % with *M. yunxiaonensis*), ANI (both strains 90.6 and 80 %, respectively) and AAI (both strains 93.1 and 80.1 %, respectively) below the recommended thresholds for prokaryotic species delineation.

The phylogenetic analysis and genome comparison support that strains UG2_1^T^ and UG2_2 represented a novel species within the genus *Mangrovimonas*, for which the name *Mangrovimonas cancribranchiae* sp. nov. is proposed.

## Genome features

The comparison of the genome features of the two strains against those of the four closest related strains of the genus *Mangrovimonas* (*M. spongiae* HN-E26^T^, *M. yunxiaonensis* LYYY01^T^, *M. futianensis* AS18^T^ and *M. aestuarii* MBT5^T^) is reported in Table S1. Strain UG2_1^T^ has a genome size of 3 085 202 bp with G+C content of 33.8 mol% and 2905 predicted genes, while strain UG2_2 had a genome with ~10 000 fewer nucleotides but the same G+C content. Both genomes are contained within a single contig. Strain UG2_1^T^ shows 2857 annotated protein-coding genes, two pseudogenes and 46 RNA genes, while UG2_2 differs only in terms of a minor number of protein-coding genes (2 840 vs 2857), as expected by the smaller genome size. Notably, the differential nucleotides present only in UG2_1^T^ form genes scattered throughout the genome. These genes encode for hypothetical proteins of unknown function or represent extra copies of other genes. Despite such differences, the elevated genetic relatedness and similarity among the two strains suggest that they are descendants of the same ancestor (i.e., clonal varieties), as also shown by the ERIC-PCR fingerprints (Fig. S4).

The UG2_1^T^ and UG2_2 genomes are larger in size than those of *M. aestuarii* (2.95 Mbp), *M. spongiae* (2.77 Mbp), and *M. yunxiaonensis* (2.64 Mbp). They shared a large proportion of conserved regions with the closest relatives *M. spongiae* (91.5 %), *M. yunxiaonensis* (79.0), *M. aestuarii* (75.5 %), and *M. futianensis* (75.2%), while the remaining portions are unique to our strains or missing from the reference genomes (Fig. S7). For instance, one proviral region of 31 000 bp, not present in the closest relatives *M. spongiae* and *M. yunxiaonensis*, has been found in the genomes of UG2_1^T^ and UG2_2, suggesting that these isolates have previously experienced a viral attack. Notably, it has been shown that prophages are beneficial for withstanding osmotic and oxidative stresses, increasing growth, and influencing biofilm formation [58], representing a possible adaptative advantage for UG2_1^T^ and UG2_2 on the crab gill.

Carbon organic catabolism genome analysis showed that both UG2-1^T^ and UG2-2 genomes have the complete pathways for the utilization of galactose and xylose among sugars, alanine, asparagine and aspartate among amino acids, fumarate as organic acid, and ethanol (Table S3). Notably, only *M. futianensis* showed the ability to catabolize a larger range of carbon sources, including arabinose, cellobiose, glucose, maltose, trehalose, succinate, and l-malate (Table S3). Further analyses were conducted to evaluate the presence of key adaptative genomic features of UG2_1^T^ and UG2_2. The two isolates possess two full-length biosynthetic gene clusters, one of which encodes a carotenoid terpene (20 738 bp) and the other a flexirubin polyketide (53 470 bp) showing different gene elements compared to *M. spongiae* and *M. yunxiaonensis* genomes (Fig. S8). Remarkably, the presence of a complete carotenoid gene cluster and the synthesis of flexirubin pigment, recently acknowledged for its antioxidant properties [59,60], suggests a potential adaptive advantage conferred to the bimodal crab host *C. inversa* by attenuating oxidative stress on gill tissues during the animal’s emersion periods [61]. Additionally, the sequences of UG2_1^T^ and UG2_2 encode for several stress-related genes potentially conferring symbiotic advantages during their association with the animal host. Notably, *gudB* (glutamate dehydrogenase), *glnA* (glutamine synthetase), *qorA* and *qorB* (quinone oxidoreductase), *uvrA*, *uvrB*, *uvrC* (UvrABC system protein), *yaaA* (peroxide stress resistance protein) and *cspC* (cold shock-like protein) were detected. Genes encoding glutamine synthetase and quinone oxidoreductase have already been detected by metagenomic analyses of gill microbial communities in different fiddler crab species across two continents [12]. They were indicated as possible players in the ammonia metabolism on the gill surface, serving as a detoxification system for the crab in the absence of water. From a coevolutionary perspective, the stable partnership with bacteria capable of producing antioxidant pigments and catabolite recycling (e.g., ammonia and carbon dioxide) has been hypothesized as a critical milestone in brachyuran crabs’ terrestrialization, supporting the gill functioning and increasing the host’s resilience outside of the water (10–12,15]. At the same time, the presence of *UvrABC* system proteins may function as a natural defence mechanism against the deleterious effects of ultraviolet radiation in combination with that of pigments (i.e., carotenoids and flexirubin).

In this context, the isolation of bacteria associated with gills, as the here proposed *M. cancribranchiae*, represents an essential tool for manipulative experiments that could provide deeper insights into the evolutionary process of terrestrial adaptation in brachyuran crabs.

## Phenotypic and physiological features

The cultural characteristics of strains UG2_1^T^ and UG2_2 were determined in MB (or on MA) after 3 days of growth at 30 °C. The Gram-staining test was carried out by using a Gram-staining Kit (Volu-Sol), and the cell morphology was observed under a DM4000 B LED optical microscope (Leica) and a teneo scanning electron microscope (FEI). Gliding motility was determined following the method described by Bowman [62]. Briefly, we used ¼ strength MB supplemented with 1 % agarose and observed motility under a phase-contrast light microscope (Leica). Growth at different temperatures (4, 10, 20, 25, 28, 30, 35, 37, 40, and 45 °C) was tested on MA. Growth at different pH values was tested at 30 °C in MB, in a pH range of 4.0–12.0 at intervals of 1 pH unit, adding the appropriate biological buffers as described by Zhang and colleagues [19], specifically citrate–phosphate for the pH range 3.0–7.0, Tris–HCl for the pH range 8.0–9.0, and sodium carbonate–sodium bicarbonate for the pH range 10.0–11. Fresh MB cultures of the strains were used as inocula (50 µl) in each experiment after being standardized to contain 6×10^8^ cells ml^−1^. Results were evaluated after 7 days of incubation. NaCl tolerance for salinity was tested using a free-NaCl medium described by Li *et al*. [17] added with different NaCl concentrations (0–12 % at 1 % intervals, w/v); inoculated plates were incubated for 7 days at 30 °C. The optimal growth ranges were assessed by counting the number of growing colonies per plate on MA and the number of bacterial cells by means of a Thoma cell counting chamber in liquid MB at the end of the incubation period. Anaerobic growth was evaluated at 30 °C after 15 days on MA plates placed in an anaerobic culture jar (Oxoid that has been filled with a gas mixture of 95 % N_2_ and 5 % H_2_ in an anaerobic cabin (Glovebox Airlock, Coy Lab Products).

The type strains *M. spongiae* HN-E26^T^ (=MCCC 1K03326^T^), *M. yunxiaonensis* LYYY01^T^ (=CGMCC 1.12280^T^), and *Meridianimaribacter flavus* NH57N^T^ (=MCCC 1A03544^T^) were used as references to perform the subsequent enzymatic activity, hydrolysis, carbon source utilization, acid production and antibiotic susceptibility tests, as well as chemotaxonomic characterization, together with the two novel isolates. The strains were obtained from the Belgian Coordinated Collections of Microorganisms (BCCM/LMG) bacteria catalogue (LMG30458, LMG27142 and LMG24839, respectively) and grown following the recommended culture conditions on MA/MB media unless otherwise stated.

Oxidase and catalase activities were detected through oxidase reagent (bioMérieux) and bubble production after adding a 3 % (v/v) hydrogen peroxide solution. Hydrolysis of Tween 40, Tween 80, starch, and casein were tested on MA media with a final concentration of the substrates of 1 % (w/v). Additional physiological and biochemical characteristics (i.e., acid production and enzyme activities) were tested by using API 20NE (ampules from 1 to 8), API ZYM and API 50CH strips (bioMérieux) following the manufacturer’s instructions. The API 20NE, API ZYM and API 50CH strips were read after incubation at 30 °C for 48 h, 4 h and 52 h, respectively. Utilization of carbon sources was tested using the API 20NE strip (ampules from 9 to 20) and Phenotype Microarray Biolog PM1 and PM2 plates using a modified MB prepared without adding peptone and yeast extract or filtered seawater. Inoculation and incubation of the strips and plates were done according to the manufacturer’s instructions at 30 °C. Antibiotic resistance to amoxicillin (10 µg), chloramphenicol (30 µg), cycloheximide (10 µg), doxycycline (10 µg), kanamycin (30 µg), rifampicin (5 µg), streptomycin (10 µg) and tetracycline (30 µg) was tested using the solid agar diffusion approach described by Barry [63]. The strains were inoculated on MA in which circular wells (diameter 10 mm) had been cut for the addition of antibiotics (0.1 ml), and the diameter of the inhibited growth zone was measured after incubation at 30 °C for 3 days. Assessment of flexirubin pigments was observed by the colour change of suspending cells after the addition of 20 % (w/v) potassium hydroxide solution [64]. The indole test was carried out by growing the strains and *Escherichia coli* as a positive control in tryptone water for 36 h and adding to each tube 10 % (v/v) of Kovac’s reagent, which, in the presence of indole, turned its colour to deep red. The nitrogen reduction test was performed using the homonymous commercial kit from Sigma Aldrich following the producers’ instructions.

The growth features and key phenotypical characteristics of the proposed strains compared with those of the reference species are presented in Table 1.

**Table 1.** Differential phenotypic characteristics of UG2_1^T^, UG2_2 and related type strains Strains: 1, UG2_1^T^; 2, UG2_2; 3, *Mangrovimonas spongiae* HN-E26^T^ (=MCCC 1K03326^T^); 4, *Mangrovimonas yunxiaonensis* LYYY01^T^ (=CGMCC 1.12280^T^); 5, *Meridianimaribacter flavus* NH57N^T^ (=MCCC 1A03544^T^). All data are from this study unless otherwise indicated. +, Positive; w, weakly positive; −, negative; S, susceptible; R, resistant. When test results are negative for all six strains, they are not reported.

Physiology	1	2	3	4	5
Growth range (optimum):					
Temperature (°C)	20–37 (28–35)	20–37 (28–35)	10–40 (30)*	10–38 (30–37)^†^	9–37 (30–37)^‡^
pH	5–9 (6–7)	5–9 (6–7)	6–9 (7)*	6–10 (7–9)^†^	6.5–8.5 (7.8)^‡^
Salinity (%)	1–11 (2–4)	1–11 (2–4)	0.5–12 (4–7)*	1–7 (2–5)^†^	0.5–4.0 (0.5–2.0)^‡^
Colony colour	Orange	Orange	Orange	Orange	Yellow
Flexirubin production	+	+	+	+	−
Hydrolysis of:					
Casein	+	+	−	−	−
Tween 80	+	+	−	+	−
Starch	−	−	+	−	+
Utilization of (API 20NE):					
Maltose	−	−	−	w	−
Enzyme activity (API ZYM):					
Esterase (C4)	−	−	+	+	−
Esterase lipase (C8)	−	−	+	−	−
Leucine arylamidase	+	+	−	+	+
Acid phosphatase	−	−	+	+	−
Acid formation from (API CH50):					
Potassium 5-ketogluconate	+	+	−	−	−
Cellobiose	−	−	−	−	+
Maltose	−	−	−	−	+
Lactose (bovine origin)	−	−	−	−	+
d-Glucose	−	−	−	−	+
Amygdalin	−	−	−	−	w
Starch	−	−	−	−	w
Aesculin in ferric citrate	+	+	w	+	+
Susceptibility to:					
Chloramphenicol	S	S	S	S	R
Tetracycline	S	S	S	S	R

*Data from Zhuang *et al*. [17]. †Data from Li *et al*. [16]. ‡Data from Wang *et al*. [67].

Strains UG2_1^T^ and UG2_2 grew well on MA, and all colonies were smooth, circular, translucent, light orange to orange in colour, shiny with entire margins and 1–2 mm in diameter after incubation at 30 °C for 3–4 days (Fig. 1e). The two strains were Gram-stain-negative and strictly aerobic. Under both optical and scanning electron microscope observations, the cells were rod-shaped, 1.0–2.3 µm long and 0.3–0.5 µm in diameter, motile by gliding with no flagella (Fig. 1f, g). Growth occurred at temperatures ranging from 20 to 37 °C (optimum, 28–35 °C), at pH 5.0–8.0 (optimum, pH 6.0–7.0) and in the presence of 1–11 % (optimum, 2–4 %, w/v) NaCl. Production of flexirubin pigment was positive for all *Mangrovimonas* strains but not for *Meridianimaribacter flavus*. All strains were positive for the activity of oxidase, catalase, and alkaline phosphatase, while they were negative for nitrate reduction and indole production tests. Gelatine of bovine origin and aesculin ferrum citrate (from API 20NE test), along with Tween 40, were hydrolysed by all the strains. Our strains were also able to hydrolyse Tween 80 and casein, but not starch, as observed for *M. spongiae* and *M. flavus*. Results for the assimilation of sugars available in the API 20NE (i.e., d-glucose, l-arabinose, d-mannose, d-mannitol, *N*-acetyl-glucosamine, potassium gluconate, capric acid, adipic acid, malic acid, trisodium citrate and phenylacetic acid) showed that UG2_1^T^, UG2_2, *M. spongiae* HN-E26^T^, and *Meridianimaribacter flavus* NH57N^T^ were not able to use any of these as a sole carbon source, while *M. yunxiaonensis* was able to grow on maltose weakly. Similarly, in the condition tested (MB without peptone and yeast extract or filtered seawater), our strains were not able to use any of the substrates in the PM1 and PM2 plates as the sole carbon source. Such limitation in using sole carbon source in *in vitro* tests was also documented for other *Mangrovimonas* type species in our experiments and by other authors [16,19]. However, the metabolic pathway analysis from the genome sequences and the predicted proteomes supports the potential of UG2_1^T^ and UG2_2 to utilize different carbon sources, including sugars, amino acids, and organic acids (Table S3). Thus, given that (i) the genes encoding these pathways are present in the genomes and (ii) the strains grow well in the presence of peptone and yeast extract in the media (i.e., MB), we can attribute the lack of growth in the carbon-depleted media to the need for various compounds essential for bacterial growth and metabolism, such as amino acids, peptides, vitamins, minerals, and cofactors [65,66], that are present in the peptone and yeast extract.

As per enzymatic activities detected through the API ZYM kit, all strains were negative for the activity of lipase (C14), valine arylamidase, cystine arylamidase, trypsin, naphthol-AS-BI-phosphohydrolase, α-chymotrypsin, α-galactosidase, β-galactosidase, β-glucuronidase, α-glucosidase, β-glucosidase, *N*-acetyl-β-glucosaminidase, α-mannosidase and α-fucosidase. Both isolates were found to be negative for esterase (C4), esterase lipase (C8) and acid phosphatase activities, while the closest relative *M. spongiae* was positive. On the contrary, leucine arylamidase activity is present in UG2_1^T^ and UG2_2, *M. yuxiaoensis* and *M. flavus* but not in *M. spongiae*. As indicated by the API 50CH strips, all the strains were unable to form acids from erythritol, d-arabinose, l-arabinose, d-ribose, d-xylose, l-xylose, d-adonitol, methyl β-d-xylopyranoside, d-galactose, d-fructose, d-mannose, l-sorbose, l-rhamnose, inositol, dulcitol, d-mannitol, d-sorbitol, methyl α-d-mannopyranoside, methyl α-d-glucopyranoside, *N*-acetylglucosamine, arbutin, aesculin, salicin, melibiose, sucrose, trehalose, inulin, melezitose, raffinose, glycogen, xylitol, gentiobiose, turanose, d-lyxose, d-tagatose, d-fucose, l-fucose, d-arabitol, l-arabitol, potassium-gluconate and potassium-2keto-gluconate. UG2_1^T^ and UG2_2 were the only strains found to be positive for acid production from potassium 5-ketogluconate. As the other *Mangrovimonas* reference strains, UG2_1^T^ and UG2_2 were unable to form acids from cellobiose, maltose, lactose, d-glucose, amygdalin and starch, while *Meridianimaribacter flavus* showed a pH decrease in the presence of those substrates. Antibiotic susceptibility tests showed that all the strains were susceptible to doxycycline, rifampicin, and amoxicillin but resistant to kanamycin, streptomycin and cycloheximide. The only difference was noted in *M. flavus* being resistant to chloramphenicol and tetracycline.

Overall, the phenotypic and physiological features of UG2_1^T^ and UG2_2 were consistent with a single species and clearly distinguished them from other closely related strains.

## Chemotaxonomic characteristics

For the chemotaxonomic characterization, UG2_1^T^, UG2_2, and three closely related type strains were cultured on MB at 30 °C for 3 days. The biomass of all strains was centrifuged and harvested at 5000 *g* for 15 min for the analysis of cellular fatty acids, respiratory quinones, and polar lipids. All analyses of chemotaxonomic characteristics were carried out by the identification service of DSMZ (Leibniz-Institut DMSZ – Deutsche Sammlung von Mikroorganismen und Zellkukturen GmbH, Braunschweig, Germany).

As shown in Table 2, UG2_1^T^ and UG2_2 contained iso-C_15 : 0_ (36.9–37.3 %), iso-C_15 : 1_ G (25.8–27.8 %), iso-C_17 : 0_ 3OH (7.4–12.0 %), iso-C_15 : 0_ 3OH (6.0–7.1 %), C_15 : 0_ aldehyde (5.7–9.2 %), C_15 : 0_ (3.8 %), summed feature 3 (identified as iso-C_15 : 0_ 2-OH and C_16 : 1_* ω*7*c*; 1.7 %), C_16 : 0_ 3OH (0.8–1.1 %) and other trace components. The compositions of cellular fatty acids were mostly similar to the three closely related type strains, except for lower C_15 : 0_ compared to *M. spongiae* (9.8 %), higher iso-C_15 : 0_ 3OH compared with *M. spongiae* (3.3 %) and *M. yunxiaonensis* (4.6 %), and higher iso-C_17 : 0_ 3OH compared with *M. spongiae* (3.8 %), *M. yunxiaonensis* (3.1 %) and *M. flavus* (4.9 %). The main respiratory quinone of UG2_1^T^ and UG2_2 was MK6 (97.5–98.7 %), followed by MK5 (1.1–2.3 %) and MK7 (0.2 %), which was consistent with the closely related species. The only differences that could be noted were that MK5 was not identified in *M. spongiae*, *M. yunxiaonensis* and *M. flavus*, and MK7 was not found in *M. yunxiaonensis*. The polar lipids of UG2_1^T^ and UG2_2 comprised phosphatidylethanolamine, one unidentified glycolipid, one unidentified phospholipid, two unidentified aminolipids, and four unidentified lipids (Fig. S9). Compared with the three related type strains, the main difference was that phospholipid was not identified in *M. yunxiaonensis*.

**Table 2.** Cellular fatty acid compositions (%) of UG2_1^T^, UG2_2 and related type strains Strains: 1, UG2_1^T^; 2, UG2_2; 3, *Mangrovimonas spongiae* HN-E26^T^ (=MCCC 1K03326^T^); 4, *Mangrovimonas yunxiaonensis* LYYY01^T^ (=CGMCC 1.12280^T^); 5, *Meridianimaribacter flavus* NH57N^T^ (=MCCC 1A03544^T^). The cellular fatty acid profiles have been identified using the Type Strain Fatty Acid Database (TSBA) version 6 within the Microbial Identification System. Only components that have at least 1 % in one strain have been listed. Major components (>5.0 %) are highlighted in bold; tr, trace amount (<0.5 %); −, not detected.

Fatty acid	1	2	3	4	5
**Saturated**					
C_14 : 0_	tr	0.5	1.0	0.6	tr
C_15 : 0_	3.8	3.8	**9.8**	4.7	1.8
C_16 : 0_	1.0	0.9	1.9	2.0	0.6
**Unsaturated**					
C_15 : 1_ ω11*c*	–	–	2.3	–	–
**Branched chain**					
iso-C_13 : 0_	0.8	1.1	0.6	1.7	0.5
iso-C_14 : 0_	–	–	1.1	4.1	0.6
iso-C_15 : 0_	**37.3**	**36.9**	**31.8**	**36.2**	**28.3**
iso-C_16 : 0_	tr	tr	1.2	3.2	tr
anteiso-C_15 : 0_	0.6	0.8	1.3	2.7	1.2
iso-C_15 : 1_ G	**25.8**	**27.8**	**24.2**	**18.7**	**31.7**
iso-C_15 : 0_ 2OH	–	–	0.6	0.6	0.5
iso-C_15 : 0_ 3OH	**7.1**	**6.0**	3.3	4.6	**7.3**
iso-C_16 : 0_ 3OH	0.5	tr	1.1	1.7	2.0
iso-C_16 : 1_ h	–	–	–	1.4	–
iso-C_17 : 0_ 3OH	**12**	**7.4**	3.8	3.1	4.9
**Hydroxy**					
C_16 : 0_ 3OH	1.1	0.8	0.9	0.6	tr
**Aldehyde**					
C_15 : 0_ aldehyde	**5.7**	**9.2**	**8.2**	**8.1**	**9.7**
Summed feature*					
3†	1.7	1.7	4.0	1.2	**8.1**
9‡	–	–	–	2.2	–

*Summed Ffeatures are fatty acids that cannot be resolved reliably from another fatty acid using the chromatographic conditions chosen. The MIDImidi system groups these fatty acids together as one feature with a single percentage of the total.

†Summed Feature 3, iIdentified as iso-C_15 : 0_ 2-OH and C_16 : 1_* ω*7*c* in strains 1, 2, 3, 4 and as only iso-C_15 : 0_ 2-OH in strain 5.

‡Summed Feature 9, iIdentified as iso-C_17 : 1_* ω*7*c.*

## Description of *Mangrovimonas cancribranchiae* sp. nov.

*Mangrovimonas cancribranchiae* (can.cri.bran’chi.ae. L. masc. n. *cancer*, a crab; L. fem. n. *branchia*, gill; N.L. gen. n. *cancribranchiae*, of the gill of a crab).

Cells are Gram-stain-negative, strictly aerobic, rod-shaped, 1.0–2.3 µm long and 0.3–0.5 µm wide. Colonies are smooth, circular, translucent orange, shiny with entire margins and 1–2 mm in diameter after incubation on MA at 30 °C for 3 days. Flagella are not found. Gliding motility is observed. Growth occurs at 20–37°C (optimum, 28–35 °C), at pH 5.0–8.0 (optimum, pH 6.0–7.0) and with 1–11 % NaCl salinity (optimum, 2–4 %, w/v). Cells produce the pigment flexirubin. Nitrate is not reduced, and indole production is not present. Cells are positive for hydrolysis of Tween 40, Tween 80, casein, and gelatin but negative for starch. The carbon compounds (*n*=190) present in the Biolog PM1 and PM2 systems, including 70 carbohydrates, 60 carboxylic acids, 30 amino acids, 11 polymers, six alcohols, five amines, three amides, and five fatty acids, are not utilized as sole carbon sources for growth and active metabolism. Acid production from carbohydrates is negative except for potassium 5-keto-gluconate. Cells are positive for the activity of oxidase, catalase, leucine arylamidase and alkaline phosphatase, but negative for esterase (C4), esterase lipase (C8), lipase (C14), cystine arylamidase, trypsin, α-chymotrypsin, α-galactosidase, β-galactosidase, β-glucuronidase, α-glucosidase, β-glucosidase, *N*-acetyl-β-glucosaminidase, α-mannosidase, α-fucosidase, valine arylamidase, acid phosphatase, and naphthol-AS-BI-phosphohydrolase. Susceptible to rifampicin, tetracycline, amoxicillin, chloramphenicol, and doxycycline but resistant to kanamycin, streptomycin, and cycloheximide. The predominant cellular fatty acids contain iso-C_15 : 0_, iso-C_15 : 1_ G, iso-C_17 : 0_ 3OH, and iso-C_15 : 0_ 3OH. The main respiratory quinone is MK6. The major polar lipids are phosphatidylethanolamine, one unidentified glycolipid, one unidentified phospholipid, two unidentified aminolipids, and four unidentified lipids.

The type strain, UG2_1^T^ (=KCTC 102158^T^=DSM 117025^T^), was isolated from the gills of crab *Cranuca inversa* in the mangrove forest of KAUST Ibn Sina Field Research Station, Thuwal, Saudi Arabia. The GenBank accession numbers for the 16S rRNA gene and complete genome sequences are OR538380 and CP136925 (BioProject PRJNA1023480), respectively.

## supplementary material

10.1099/ijsem.0.006415 Uncited Supplementary Material 1.

## Abbreviations

AAI: average aminoacidic identity
ANI: average nucleotide identity
dDDH: digital DNA–DNA hybridization
MA: marine broth agar
MB: marine broth
TYGS: Type (Strain) Genome Server
