# Supplementary material for: Mangrovimonas cancribranchiae sp. nov., a novel bacterial species associated with the gills of the fiddler crab Cranuca inversa (Brachyura, Ocypodidae) from Red Sea mangroves
Source: Int J Syst Evol Microbiol. 2024 Jun 12;74(6):006415. doi: 10.1099/ijsem.0.006415 (PMC11261673; doi:10.1099/ijsem.0.006415)
Supplement: Uncited Supplementary Material 1. [file ijsem-74-06415-s001.pdf]

Supplementary Material of

Mangrovimonas cancribranchiae sp. nov., a novel bacterial species associated with the gills of the fiddler crab *Cranuca inversa* (Brachyura, Ocypodidae) from Red Sea mangroves

Xinyuan Yang[1]*, Elisa Garuglieri[1]*, Marc W. Van Goethem[1], Ramona Marasco[1], Marco Fusi[2], Daniele Daffonchio[1†]

[1]Red Sea Research Center (RSRC), Biological and Environmental Sciences and Engineering Division (BESE), King Abdullah University of Science and Technology (KAUST), Thuwal, 23955-6900, Saudi Arabia

[2]Dove Marine Laboratory, School of Natural and Environmental Sciences Newcastle University, Newcastle-Upon-Tyne, NE1 7RU, United Kingdom

***Equally contribution:** X.Y. and E.G. contributed equally to this work

[†]**Correspondence:** Daniele Daffonchio; email: daniele.daffonchio@kaust.edu.sa

Supplementary Table 1. Genome characteristics of UG2_1^T, UG2_2, and the closest related type strains. Strain: 1, UG2_1^T (= CP136925); 2, UG2_2 (= CP136924); 3, *Mangrovimonas spongiae* HN-E26^T (= GCF_003944795; Zhuang et al., 2020); 4, *M. yunxiaonensis* LYYY01^T (= GCF_000733475; Li et al., 2013); 5, *M. aestuarii* MBT5^T (= GCF_028767185; Zhang et al., 2023); 6, *M. futianensis* AS18^T (= GCF_021245805; Yao et al., 2022).

**Characteristics**	**1**	**2**	**3**	**4**	**5**	**6**
Total length (bp)	3,085,202	3,072,522	2,776,420	2,641,972	2,952,053	3,699,592
Contig *N50* (bp)	3,085,202	3,072,522	635,097	482,503	313,483	410,776
Contigs	1	1	9	17	15	112
G+C (mol%)	33.8	33.8	34.0	39.2	36.5	36.1
Genes	2,905	2,888	2,533	2,446	2,653	3,374
Protein-coding genes	2,857	2,840	2,481	2,390	2,623	3,289
rRNA (5S, 16S, 23S)	6	6	5	8	4	3
tRNA	39	39	37	34	41	38
ncRNA	1	1	4	4	0	0
Pseudo Genes	2	2	6	8	0	0

Supplementary Table 2: Results of the digital DNA–DNA hybridisation (dDDH) and the average nucleotide identity based on the average nucleotide identity based on reciprocal best hits (ANI), BLAST (ANIb) and MUMmer (ANIm) algorithms, and amino acid identities (AAI) of UG2_1^T, UG2_2 and related type strains. Strain: 1, UG2_1^T (= CP136925); 2, UG2_2 (= CP136924); 3, *Mangrovimonas spongiae* HN-E26^T (= GCF_003944795.1); 4, *M. yunxiaonensis* LYYY01^T (= GCF_000733475.1); 5, *M. aestuarii* MBT5^T (= GCF_028767185.1); 6, *M. futianensis* AS18^T (= GCF_021245805; Yao et al., 2022). The boundary values proposed for species delineation are ANIb and AAI of 95% and dDDH of 70% (Richter et al., 2009; Richter et al., 2016; Rodriguez-R et al., 2016; Tu et al., 201 6; Kostaninidis 2017).

Index		1	2	3	4	5	6
dDDH (%)	1	-					
	2	100	-				
	3	43.5	43.5	-			
	4	20.8	20.8	21	-		
	5	18.2	18.2	17.8	13.6	-	
	6	18.0	17.9	17.8	18.1	17.3	-
ANI (%)	1	-					
	2	100	-				
	3	90.6	90.6	-			
	4	80.0	80.0	80.1	-		
	5	77.4	77.4	77.5	77.2	-	
	6	77.1	76.8	76.9	76.9	77.3	-
ANIb (%)	1	-					
	2	100	-				
	3	90.96	90.96	-			
	4	77.62	77.52	77.47	-		
	5	71.11	71.1	71.18	70.55	-	
	6	71.78	71.78	71.83	70.95	69.98	-
ANIm (%)	1	-					
	2	99.99	-				
	3	91.58	91.58	-			
	4	83.71	83.71	83.98	-		
	5	83.81	83.81	83.75	83.82	-	
	6	83.34	83.34	83.01	84.32	83.79	-
AAI (%)	1	-					
	2	100.0	-				
	3	93.14	93.14	-			
	4	80.07	80.10	80.43	-		
	5	67.58	67.58	68.30	68.33	-	
	6	71.09	71.05	71.21	70.86	66.44	-

Supplementary Table 3. Genomic comparison of genes involved in carbon metabolism in UG2_1^{T}, UG2_2 and close relative type strains based on GapMind catabolism of small carbon compounds (Price and Arkin, 2022). "Yes" indicates a complete pathway and colour the confidence of their completeness (i.e., green = high confidence, yellow = medium confidence), while blank boxes show the lack of a complete pathway (i.e., missing one or multiple genes). Only pathways present in at least one of the strains are listed. Strain: 1, UG2_1^{T} (= CP136925); 2, UG2_2 (= CP136924); 3, *Mangrovimonas spongiae* HN-E26^{T} (= GCF_003944795.1); 4, *M. yunxiaonensis* LYYY01^{T} (= GCF_000733475.1); 5, *M. aestuarii* MBT5^{T} (= GCF_028767185.1); 6, *M. futianensis* AS18^{T} (= GCF_021245805; Yao et al., 2022).

**C compounds**		**1**	**2**	**3**	**4**	**5**	**6**
Sugar	Arabinose						Yes
	Cellobiose						Yes
	Galactose	Yes	Yes	Yes			Yes
	Glucose						Yes
	Maltose						Yes
	Trehalose						Yes
	Xylose	Yes	Yes				Yes
Amino acid	Alanine	Yes	Yes	Yes	Yes	Yes	Yes
	Asparagine	Yes	Yes	Yes	Yes	Yes	Yes
	Aspartate	Yes	Yes	Yes	Yes	Yes	Yes
	Glutamate	Yes	Yes	Yes	Yes	Yes	Yes
	Proline	Yes	Yes	Yes			
	Serine	Yes	Yes			Yes	Yes
	Threonine	Yes	Yes			Yes	Yes
Organic acid	Fumarate	Yes	Yes	Yes	Yes	Yes	Yes
	L-malate					Yes	Yes
	Pyruvate	Yes	Yes	Yes			Yes
	Succinate					Yes	Yes
Alcohol	Ethanol	Yes	Yes	Yes	Yes	Yes	Yes

Supplementary Figure 1: Neighbour-joining phylogenetic tree based on 16S rRNA gene sequences and constructed using the software MEGAX v. 11.0.10 (Tamura et al., 2021), showing the relationship among strains UG2_1^T, UG2_2 and close relatives in the family *Flavobacteriaceae*. The position of strains UG2_1^T and UG2_2 is shown in bold. *Cryomorpha ignava* 1-22^T (AF170738) was used as the outgroup. The NCBI accession numbers are given after the species name. Numbers reported along the branches represent bootstrap values based on 1,000 replicates. Bootstrap values ≥ 50% were shown. The tree scale indicates the number of substitutions per nucleotide position.

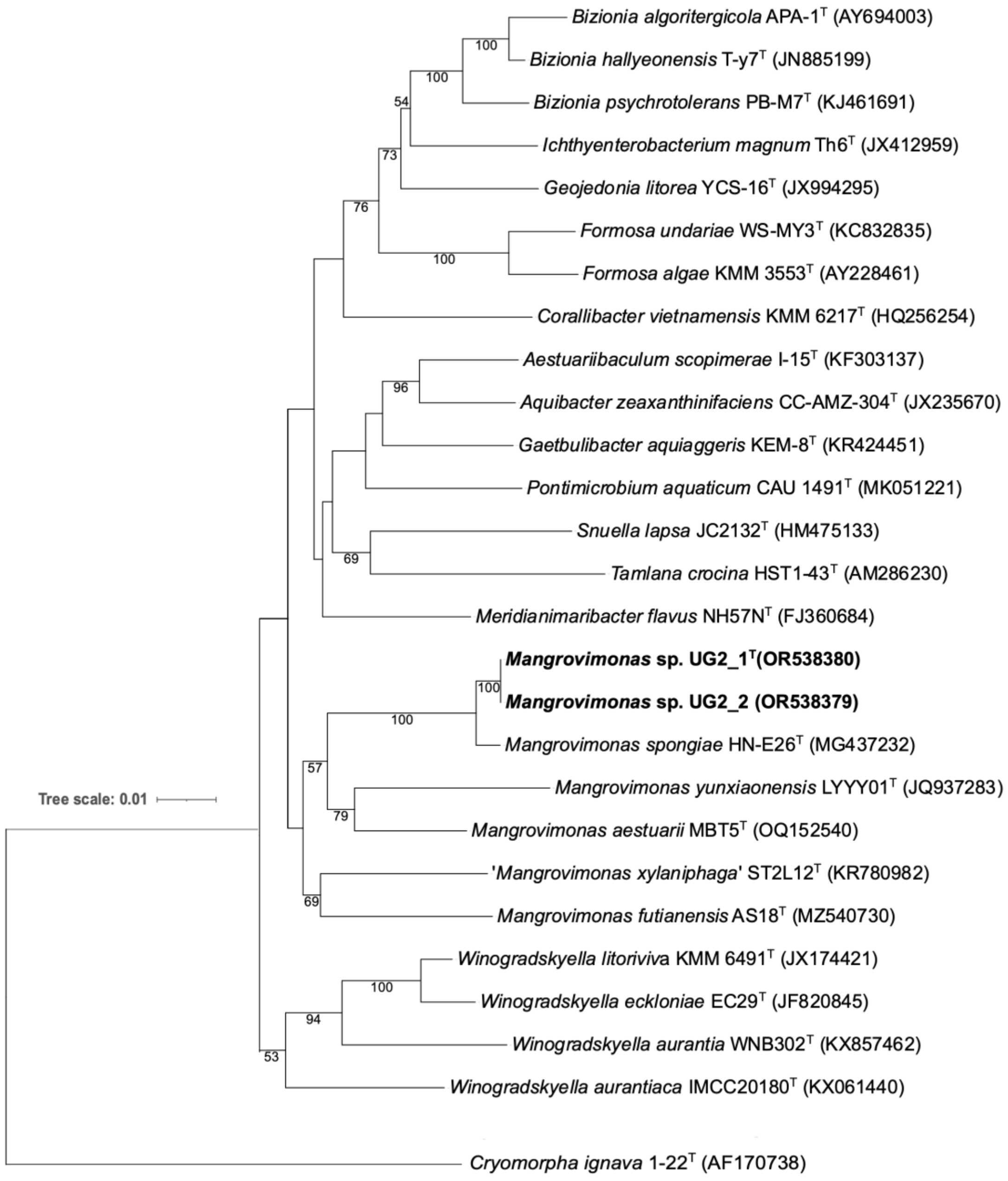

Supplementary Figure 2: Minimum evolution phylogenetic tree based on 16S rRNA gene sequences and constructed using the software MEGAX v. 11.0.10 (Tamura et al., 2021), showing the relationship among strains UG2_1^T, UG2_2 and close relatives in the family *Flavobacteriaceae*. The position of strains UG2_1^T and UG2_2 is shown in bold. *Cryomorpha ignava* 1-22^T (AF170738) was used as the outgroup. The NCBI accession numbers are given after the species name. Numbers reported along the branches represent bootstrap values based on 1,000 replicates. Bootstrap values ≥ 50% were shown. The tree scale indicates the number of substitutions per nucleotide position.

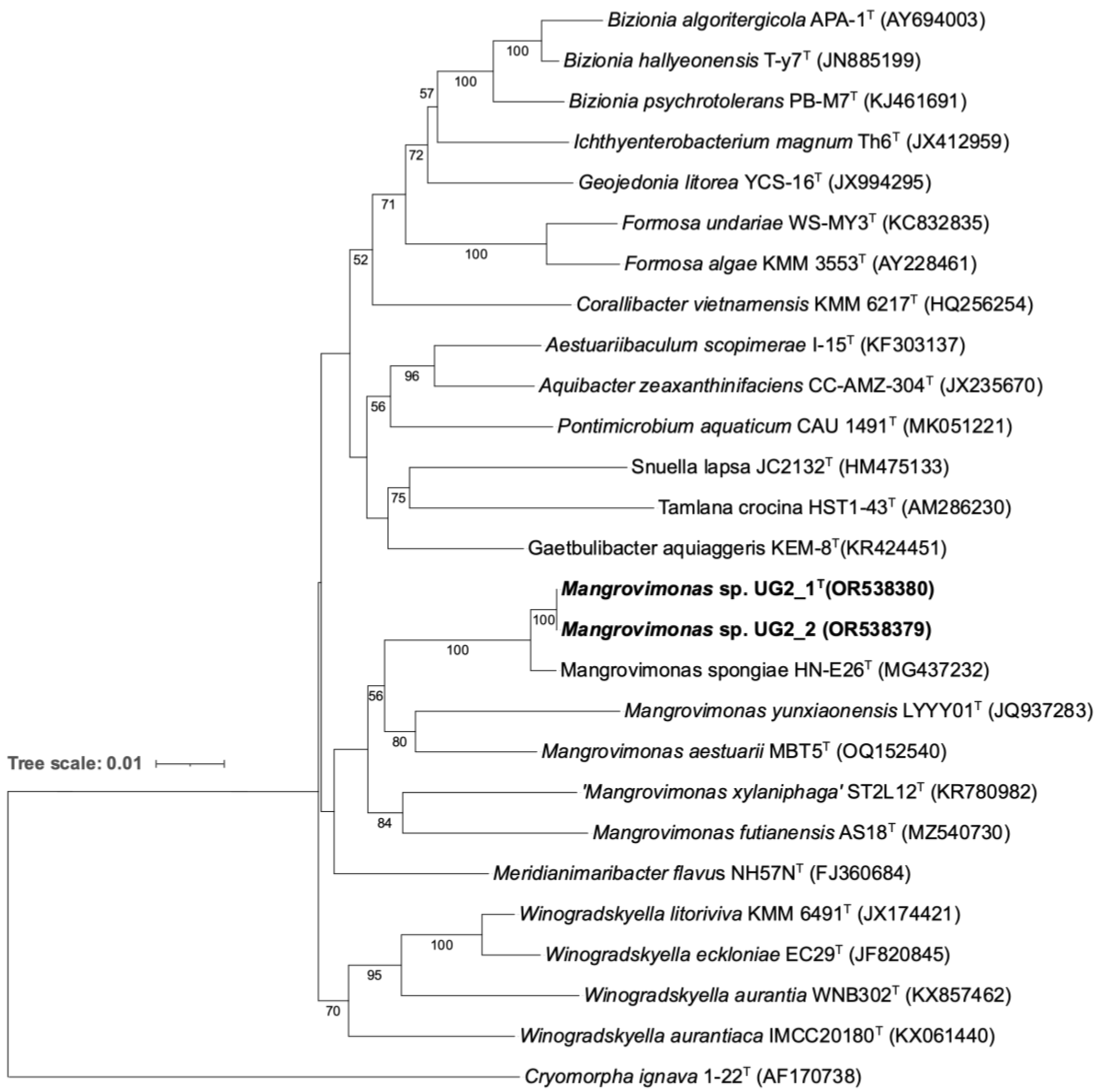

Supplementary Figure 3: Maximum parsimony phylogenetic tree based on 16S rRNA gene sequences and constructed using the software MEGAX v. 11.0.10 (Tamura et al., 2021), showing the relationship among strains UG2_1^T, UG2_2 and close relatives in the family *Flavobacteriaceae*. The position of strains UG2_1^T and UG2_2 is shown in bold. *Cryomorpha ignava* 1-22^T (AF170738) was used as the outgroup. The NCBI accession numbers are given after the species name. Numbers reported along the branches represent bootstrap values based on 1000 replicates. Bootstrap values ≥ 50% were shown.

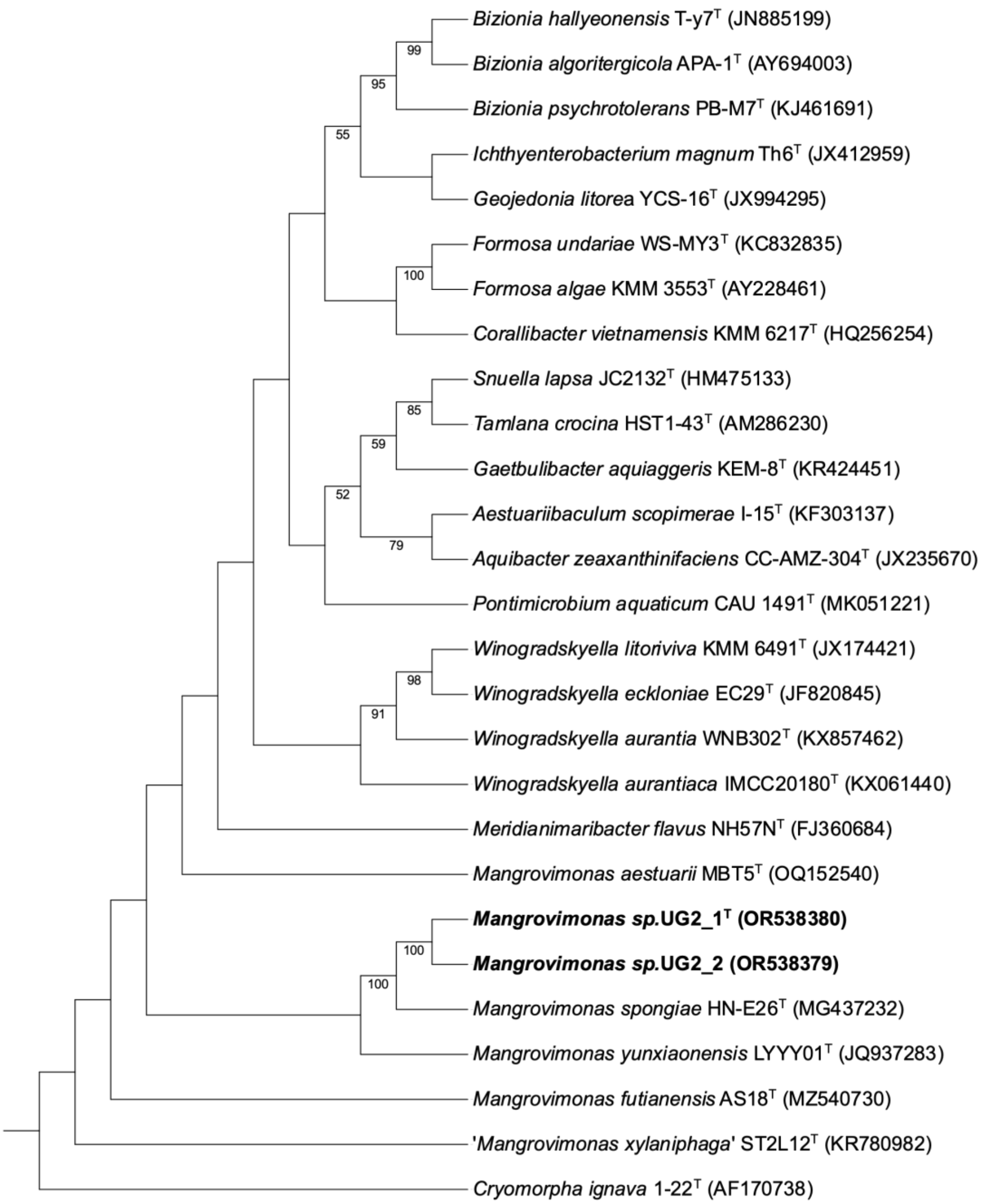

Supplementary Figure 4: Enterobacterial Repetitive Intergenic Consensus (ERIC) PCR profile of the two strains UG2_1^{T} and UG2_2 run using Bioanalyzer 2100 High Sensitivity DNA Assay. ERIC PCR was performed following the protocol described by De Bruijn et al. (1992). Three colonies per strain were selected for this analysis.

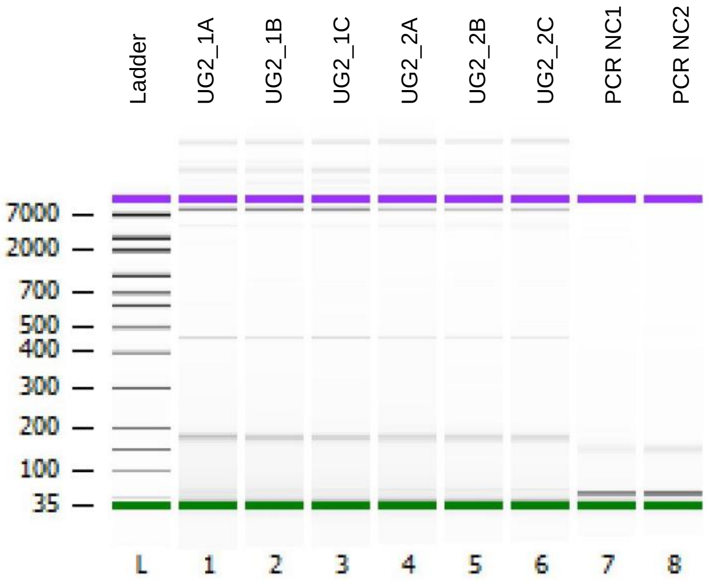

Supplementary Figure 5. Tree inferred with FastME 2.1.6.1 (Lefort et al., 2015) from GBDP distances calculated from genome sequences of UG2_1^T, UG2_2, and related type strains. The branch lengths are scaled in terms of the GBDP distance formula d5. *Hyunsoonleella pacifica* CGMCC 1.11009^T (GCA_014636335) was used as an outgroup. The NCBI accession numbers are given after the species name. The numbers above branches are GBDP pseudo-bootstrap support values > 60% from 100 replications, with an average branch support of 30.6%. (Farris, 1972). The position of strains UG2_1^T and UG2_2 is shown in bold. Colours represent different specific and sub-specific clusters the species belong to on the base of the 70% dDDH radius around each of the 17 type strains, while subspecies clustering was done using a 79% dDDH threshold. Bar represents 0.1 substitutions per nucleotide position.

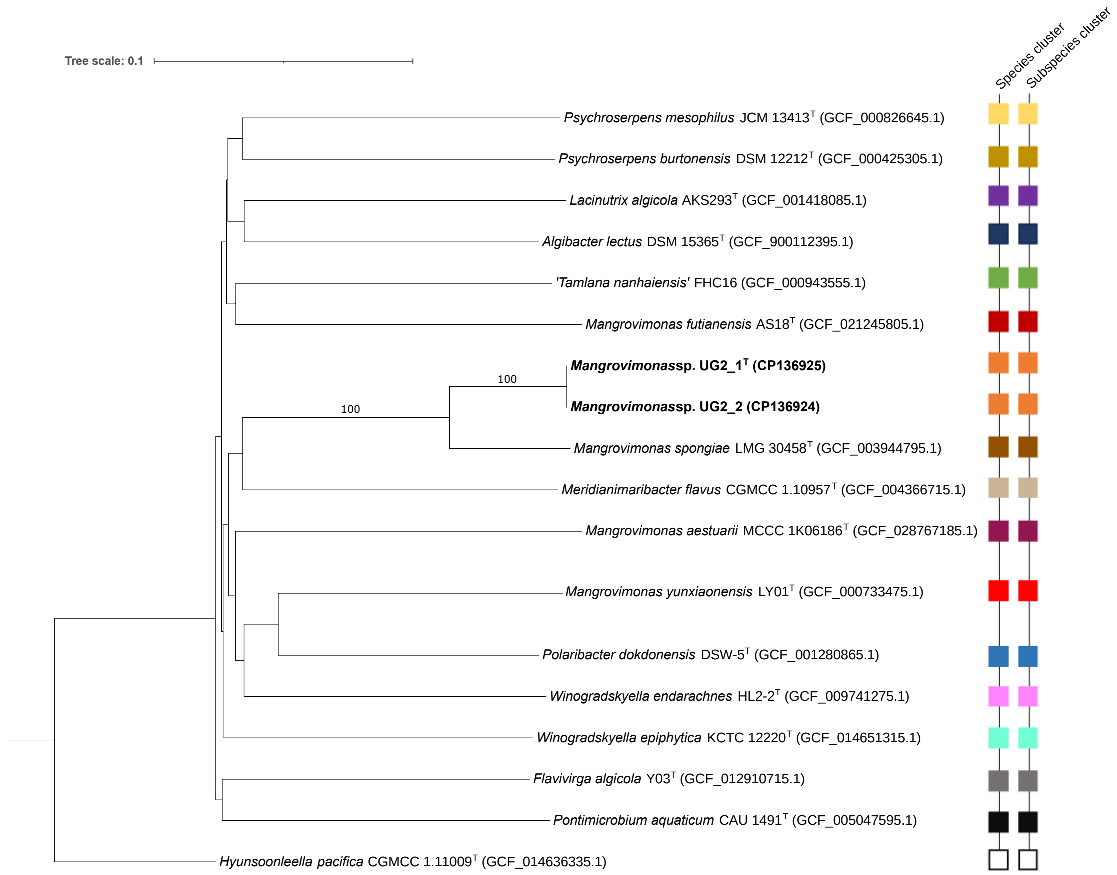

Supplementary Figure 6. Phylogenomic analysis with proposed strains UG2_1^T, UG2_2 and related taxa based on 49 concatenated single-copy genes using the maximum likelihood method made on the software FastTree2 (Price et al., 2010). The position of strains UG2_1^T and UG2_2 is shown in bold. The NCBI accession numbers are given after the species name. The tree is drawn to scale, with branch lengths measured in the number of substitutions per site. Numbers at nodes indicate bootstrap percentages using 1,000 replicates. *Robiginialea biformata* HTCC2501^T (GCF_000024125.1) was used as the outgroup. Bar, 0.1 substitutions per site.

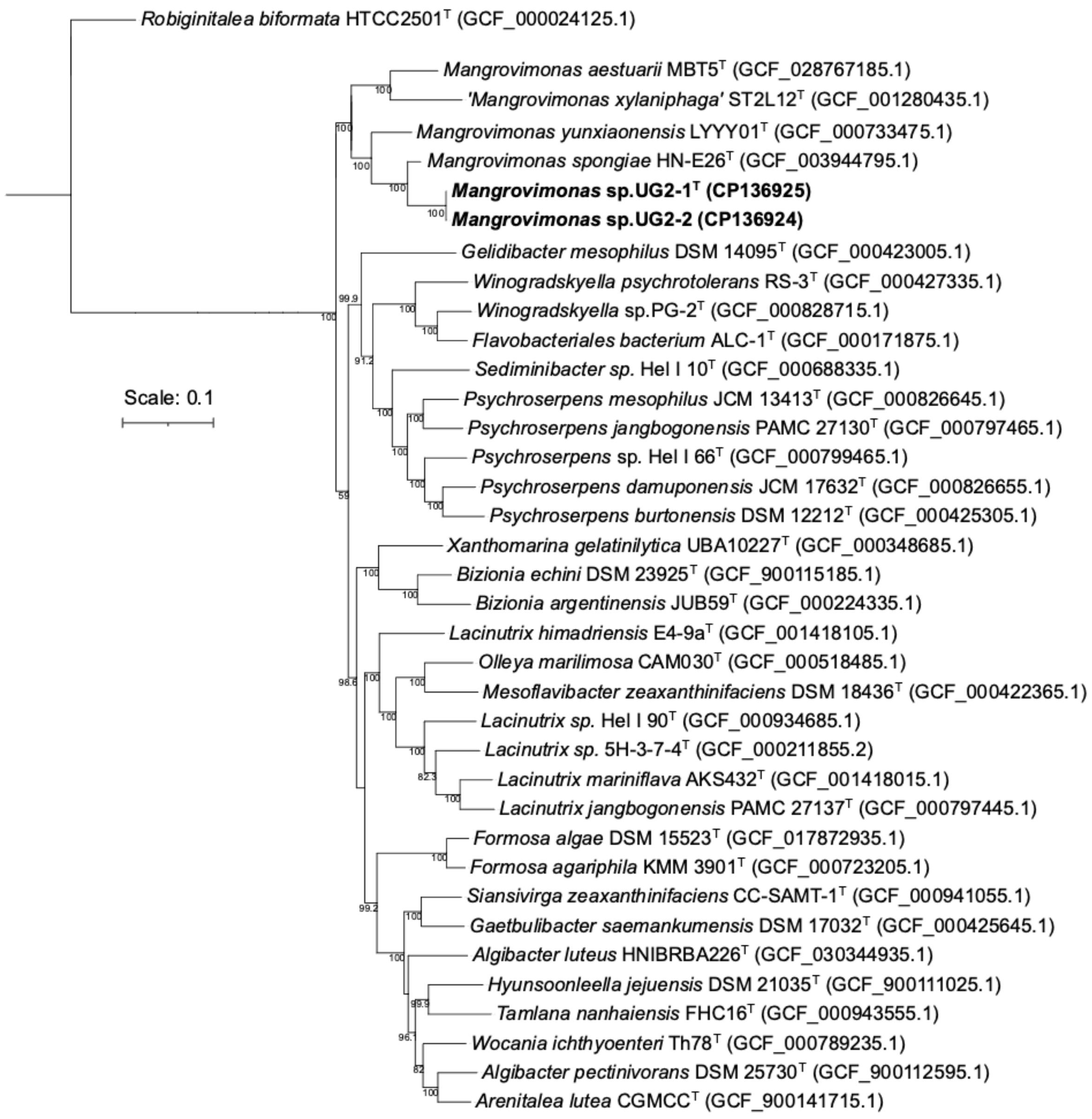

Supplementary Figure 7. Circular comparison plot showing the BLAST similarities between strain UG2_1^T (innermost grey ring) with related strains; from inside to outside (i) strain UG2_2, (ii) *M. spongiae*, (iii) *M. yunxiaonensis*, (iv) *M. aestuarii*, and (v) *M. futianensis*. The colour range corresponds to BLAST similarity scores.

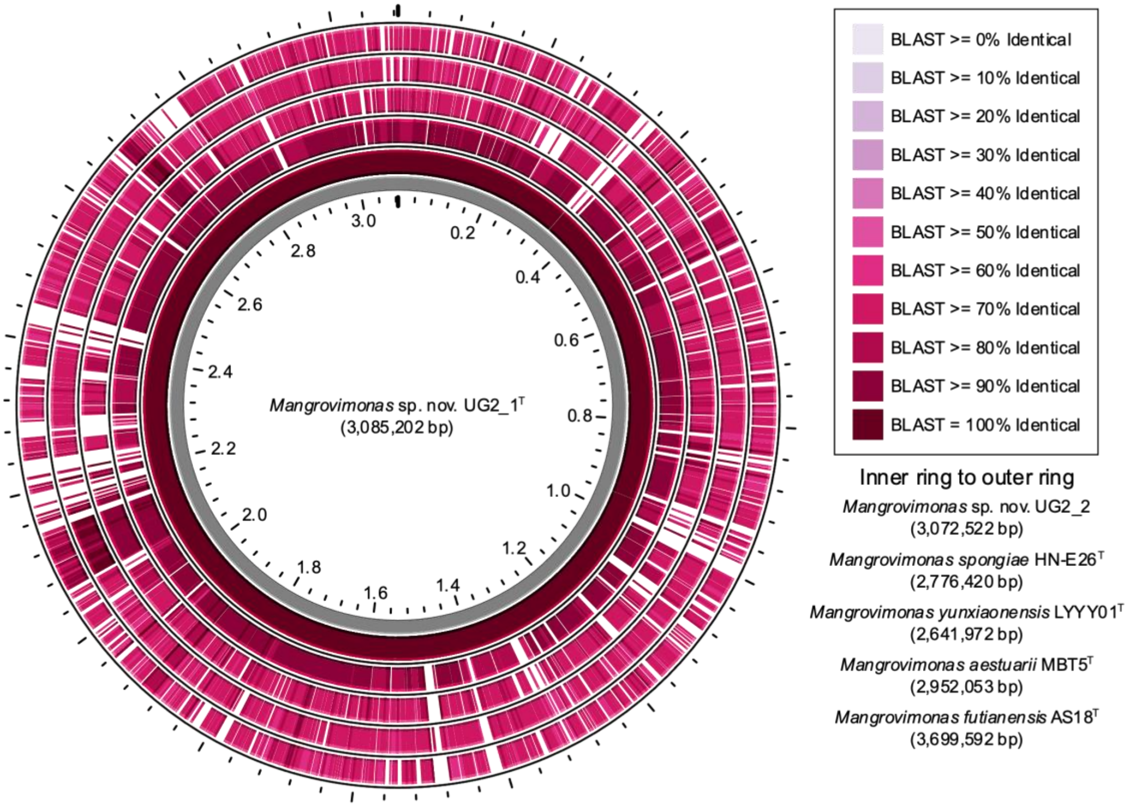

Supplementary Figure 8. Biosynthetic gene clusters (BGCs) for the synthesis of the pigments (i) flexirubin (upper part) and (ii) carotenoid (lower part) in UG2_1^{T}, UG2_2 and closest relatives. (i) The flexirubin BGC is identical in the UG2_1^{T} and UG2_2 genomes with a 53,470 bp length, while *M. spongiae* has the same length but lack the final short-chain dehydrogenase gene. *M. yunxiaonensis* (53,511 bp) has an additional regulatory gene (*MarR* family transcriptional regulator) at the start of the gene cluster, while *M. futianensis* (42,389 bp) encodes two additional regulatory genes but is truncated at the start of the BGC. *M. aestuarii* does not encode a flexirubin BGC. (ii) The carotenoid BGC (~20,800 bp) is present in all six genomes. The UG2_1^{T}, UG2_2, and *M. spongiae* genomes encode identical carotenoid BGC, while *M. yunxiaonensis* lacks amidohydrolase. *M. aestuarii* has a unique gene architecture that includes two binding sites at the start of the BGC; it lacks both the amidohydrolase and abhydrolase genes but has three additional alternative gene clusters. *M. futianensis* is the only one that includes a single binding site and a regulator gene (*Crp* family transcriptional regulator); it has two unique additional gene clusters while lacking both the amidohydrolase and abhydrolase genes.

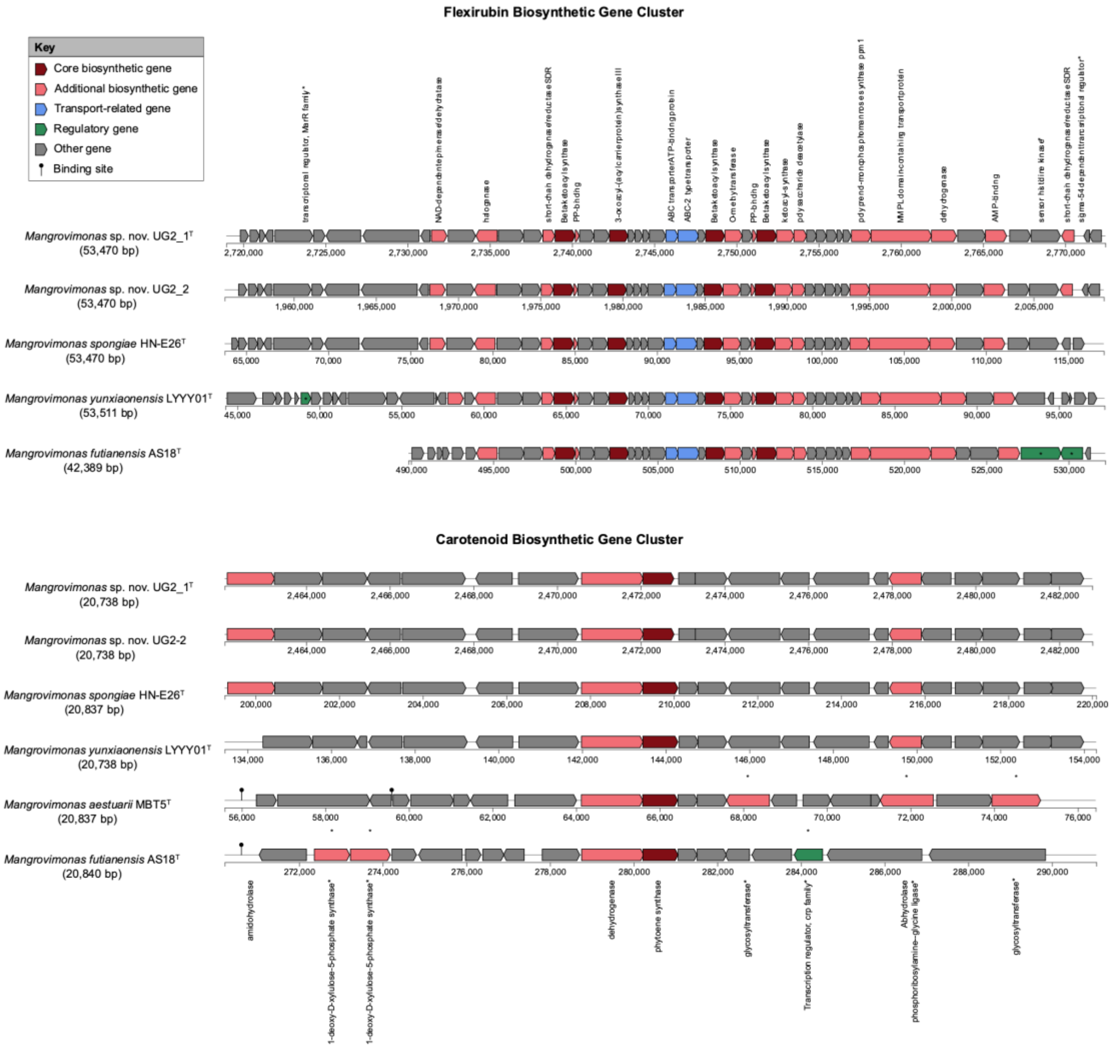

Supplementary Figure 9. Polar lipids of (**A**) UG2_1^{T}and (**B**) UG2_2, (**C**) *M. spongiae* HN-E26^T, (**D**) *M. yunxiaonensis* LYYY01^T and (**E**) *Meridianimaribacter flavus* NH57N^T separated by two-dimensional TLC, form DSMZ Identification Services polar lipid report.

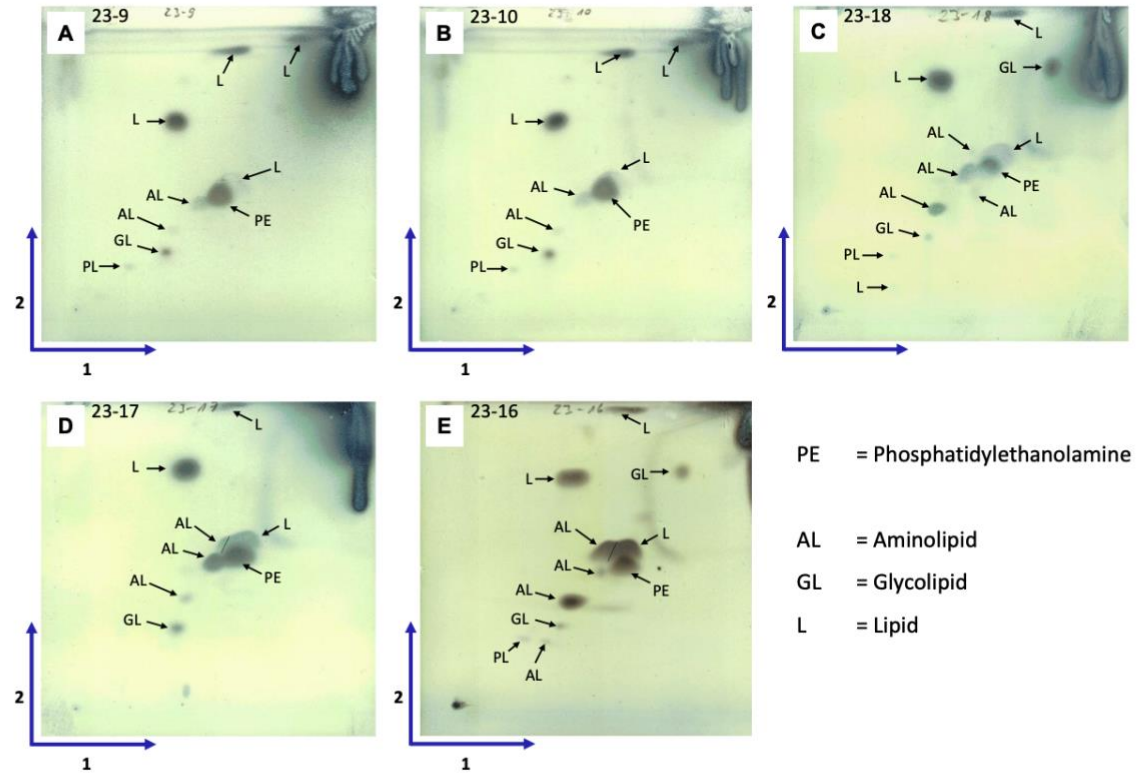

Reference

De Bruijn FJ. Use of repetitive (repetitive extragenic palindromic and enterobacterial repetitive intergeneric consensus) sequences and the polymerase chain reaction to fingerprint the genomes of *Rhizobium meliloti* isolates and other soil bacteria. *Appl Env Microb* 1992; 58:2180-7.

Farris JS. Estimating phylogenetic trees from distance matrices. *Am Nat* 1972; 106:645–667.

Konstantinidis KT, Rosselló-Móra R, Amann R. Uncultivated microbes in need of their own taxonomy. *Int J Syst Evol Microbiol* 2017; 11:2399–2406.

Li Y, Bai S, Yang C, Lai Q, Zhang H, Chen Z, Wei J, Zheng W, Tian Y, Zheng T. *Mangrovimonas yunxiaonensis* gen. nov., sp. nov., isolated from mangrove sediment. *Int J Syst Evol Microbiol* 2013; 63: 2043–2048.

Price MN, Dehal PS, Arkin AP. FastTree 2–approximately maximum-likelihood trees for large alignments. *PloS One* 2010; 5:e9490.

Price MN, Deutschbauer AM, and Arkin AP. Filling gaps in bacterial catabolic pathways with computation and high-throughput genetics. *PLoS Genetics* 2022; 18:e1010156.

Richter M, Rosselló-Móra, R, Oliver Glöckner F, Peplies, J. JSpeciesWS: a web server for prokaryotic species circumscription based on pairwise genome comparison. *Bioinformatics* 2016; 32:929–931.

Richter M, Rosselló-Móra, R. Shifting the genomic gold standard for the prokaryotic species definition. *PNAS* 2009; 106:19126–19131.

Rodriguez-R LM, Konstantinidis KT. Bypassing cultivation to identify bacterial species. *Microbe* 2014; 9:111–118.

Tamura K, Stecher G, Kumar S. MEGA11: molecular evolutionary genetics analysis version 11. *Mol Biol Evol* 2021; 38:3022–3027.

Yao S, Yang G, Zhang X, Lin C, Zhuang L. *Mangrovimonas futianensis* sp. nov., a novel species isolated from mangrove sediment. *Int J Syst Evol Microbiol* 2022; 72:005618.

Zhang W, Chen H, Lai Q, Huang Z. *Mangrovimonas aestuarii* sp. nov., isolated from tidal flat sediment. *Int J Syst Evol Microbiol* 2023; 73:006120.

Zhuang L, Lin B, Luo L. *Mangrovimonas spongiae* sp. nov., a novel member of the genus *Mangrovimonas* isolated from marine sponge. *Int J Syst Evol Microbiol* 2020; 70:1982–1986.
